# Size Does Matter: The Influence of Bulb Size on the Phytochemical and Nutritional Profile of the Sweet Onion Landrace “Premanturska Kapula” (*Allium cepa* L.)

**DOI:** 10.3390/antiox12081596

**Published:** 2023-08-10

**Authors:** Nikola Major, Nina Išić, Tvrtko Karlo Kovačević, Magdalena Anđelini, Dean Ban, Melissa Prelac, Igor Palčić, Smiljana Goreta Ban

**Affiliations:** 1 Department of Agriculture and Nutrition, Institute of Agriculture and Tourism, K. Hugues 8, 52440 Poreč, Croatia; nina@iptpo.hr (N.I.); tvrtko@iptpo.hr (T.K.K.); magdalena.andjelini@gmail.com (M.A.); dean@iptpo.hr (D.B.); melissa@iptpo.hr (M.P.); palcic@iptpo.hr (I.P.); 2 Centre of Excellence for Biodiversity and Molecular Plant Breeding, Svetošimunska 1, 10000 Zagreb, Croatia

**Keywords:** antioxidant capacity, polyphenolic compounds, sugars, FOS, landraces, Amaryllidaceae

## Abstract

The Mediterranean area is especially rich in old, both sweet and pungent, varieties of onion. The synthesis of phytochemicals takes place concurrently with the overall development and maturation of vegetables; however, it is unclear whether there is a correlation between onion bulb size and antioxidant compound content, antioxidant capacity, and nutritional parameters and what the origin of these variations is. The aim of this work was to investigate the biochemical and nutritional aspects of the sweet onion landrace “Premanturska kapula”, as well as to investigate the influence of onion bulb size on onion phytochemical and nutritional profile. The sweet onion landrace “Premanturska kapula” has a high soluble sugar content, a high antioxidant capacity, and a high phenolic compound content. Quercetin-3,4′-diglucoside and quercetin-4′-glucoside were the major flavonols, while protocatehuic acid was the major phenolic acid detected. The choice of onion bulb size can impact the profile of the sugars present, with large bulb sizes favoring higher sucrose and fructooligosaccharides content compared to small bulb sizes which were more abundant in glucose. The total sugars or bulb dry matter were not affected by bulb size. Phenolic compounds were more abundant in smaller bulb sizes, thus indicating a link between bulb development and phenolic compound allocation within the plant. This link possibly derived from agronomic practices such as bare-root transplants, or even open pollination which causes a broader genetic variability. From a consumer perspective, it can be a choice between the small and medium bulb sizes on one hand, which are more abundant in polyphenolics and simple sugars, or on the other hand, the larger bulbs which are more abundant in fructooligosaccharides known to carry excellent health benefits.

## 1. Introduction

Onion (*Allium cepa* L.) is a biennial plant and the bulb that it produces serves as an overwintering stage in its life cycle [1]. The genus *Allium* is large and consists of many wild and edible species besides onion, such as garlic, leek, chive, and shallots [2].

Onion belongs to the *Amaryllidaceae* family and probably originates from Central Asia, and it is nowadays cultivated worldwide and consumed in various forms [3]. The Mediterranean is considered a secondary gene center where onions with large bulbs are widely grown [4]. Europe is especially rich in old, both sweet and pungent, varieties of onion. Old varieties have been maintained for generations by farmers or as a part of family heritage. Due to cross breeding as a result of open pollination, old onion varieties are threatened by a loss of authenticity in terms of color, shape, or quality characteristics [5]. Furthermore, with the modernization of agriculture, farmers increasingly focused on growing onion hybrids and abandoned traditional domestic varieties, leading to genetic erosion [5].

With a long history of cultivation in different areas, onions have adapted to different climates, temperatures, and photoperiods, consequently creating a wide range of varieties [5]. The onion bulb, which grows underground, ranges in shape from flat to globular to oblong, and is either white, yellow, or red in color [6]. Onions can also be classified as sweet or non-sweet/pungent. The distinctive flavor and pungency of onions, caused by organosulfur compounds, are responsible for their importance in cooking [7].

In addition to being used for flavor, onion has been used in traditional medicine for thousands of years due to its curative properties. Onion has innumerable pharmacological activities which include anticancer, antidiabetic, antibacterial, antidermatophytic, antitoxigenic, cardiovascular, and antioxidant activities [8,9]. It acts as a stimulant, diuretic, and expectorant, as well as lowers blood sugar, lipids, and cholesterol [10]. The health benefits of onion are attributed to its various bioactive compounds, such as organosulfur compounds, phenolic compounds, polysaccharides, and saponins [11].

Onion pungency is caused by enzymatic degradation of S-alk(en)yl-L-cysteine sulfoxides into other organosulfur compounds, pyruvic acid, and ammonia during tissue damage [2]. The balance between pungency intensity and sugar content determines the perception of sweetness in an onion, allowing for the classification of onions as sweet or non-sweet [7]. About 80% of onion-bulb dry matter consists of non-structural carbohydrates which include reducing glucose and fructose, non-reducing sucrose, and a series of fructans [12]. Fructans include fructo-oligosaccharides (FOS) which mainly act as reserve carbohydrates providing energy for sprouting in plants [13,14] and have a positive impact on the stability of intestinal flora in humans [15].

Onions are a rich source of dietary flavonoids and phenolic acids, which are a part of the polyphenols family and possess effective antioxidant activity and metal chelating properties [16,17,18]. Flavonols and anthocyanins are the main subclasses of flavonoids present in onion [19]. The most abundant flavonol is quercetin, which can be found in free form, as well as in conjugated form with carbohydrates, mainly as glucosides. The two dominant quercetin glucosides, quercetin 4′-glucoside and quercetin 3,4′-diglucoside, represent about 80% of the total flavonol content in onion [20], although the concentration and distribution of quercetin may significantly vary based on the different cultivars of onions [16] and the different layers of onion bulb [21,22]. Other flavonols were also detected in onion, such as kaempferol, myricetin, and isorhamnetin [19,20], where isorhamnetin 4′-*O*-glucoside was the main form of isorhamnetin found in onions [23]. Phenolic acids found in onion include protocatehuic, *p*-coumaric, ferulic, caffeic, syringic, *p*-hydroxybenzoic, and vanillic acids [24]. The red varieties of onion generally contain the highest amount of flavonols, as well as red anthocyanins in the form of glycosides of cyanidin, peonidin, and pelargonidin [18].

Bulb size and maturity are important characteristics of onion crops. Previous studies have reported that during the period of bulb development and maturation, glucose, fructose, and sucrose content increased [13,25]. Levels of these sugars vary [26] and depend on the dry matter content which increases during bulbing [27], which explains the increase in reducing and total sugars, as well as water loss, when bulbs reach maturity [25]. Fructan levels increase during bulbing, which is followed by catabolism during the growth and sprout development of the bulbs [12]. The content of flavor precursors, which is correlated with sulfur content, increases until the sprouting of the bulb and then decreases towards maturity [10]. As the bulb weight increases, pungency levels have been shown to slightly reduce, as per the dilution effect [28].

The synthesis of many phytochemicals takes place concurrently with the overall development and maturation of vegetables [29]; however, it is unclear whether there is a correlation between onion bulb size and flavonoid concentration or not. It has been reported that small onions had higher flavonoid contents than larger ones [30]. Conversely, it has been reported that the size and bulb weight of individual onions were not correlated with quercetin glucoside concentration, indicating that small bulbs contain the same concentration of quercetin as larger bulbs [31,32]. In addition to intrinsic characteristics such as different onion cultivars, other factors have an impact on the concentration of phenolic compounds in onions, for instance soil type, sun exposure, rainfall, and whether the culture is grown in a greenhouse or field [33,34].

Consumers are showing preference for locally produced crops which are characterized by particular sensory or quality characteristics, such as sweet onions. The sweet onion landrace “Premanturska kapula” is traditionally produced in a niche geographical area at the very south end of the Istrian peninsula in Croatia, Cape Kamenjak. The name, “Premanturska kapula” comes from its geographical place of origin, village “Premantura”. According to the locals, this sweet onion landrace was cultivated in the mentioned area for more than 60 years and is characterized by a sweet and mild flavor and large bulbs. It is usually grown as an over-wintering crop without irrigation (by personal communication with producers). Therefore, the aim of this work was to investigate the differences in the phytochemical and nutritional profile between small, medium, and large sized onion bulbs, as well as the biochemical and nutritional aspects of the sweet onion landrace “Premanturska kapula”.

## 2. Materials and Methods

### 2.1. Plant Material

Onion bulbs were kindly provided by a local producer situated in the area of Premantura, Istarska County, Croatia.

Twelve bulbs, without signs of pests and disease damage or physiological disorders, were selected from approximately fifty provided bulbs of each size and divided into three groups consisting of four bulbs. The groups were divided, according to bulb size, into large (with average height 67 mm, width 114 mm, weight 468 g), medium (average height 62 mm, width 94 mm, weight 298 g), and small bulbs (average height 58 mm, width 80 mm, weight 196 g).

Alongside the chemical analyses made in this study, a morphological description was also made on the same 12 bulbs, using ECPGR descriptor for *Allium* spp. [35]. Several plant characteristics were described: bulb shape (7.1.11.), bulb skin color (7.1.15.), bulb skin thickness (7.1.17.), bulb flesh color (7.1.18.), and bulb hearting (7.1.27.).

Bulb shape was described as flat or flat globe (50% and 50%); bulb skin color was mostly a mixture of violet and brown color (66% of bulbs regardless of size); bulb skin thickness was thin (100%); the observed bulb flesh color was a mixture of violet and white in all bulbs (100%); and 92% of observed bulbs had 2–3 bulb hearts. Furthermore, bulbs were weighed, and bulb height and diameter were measured using a digital caliper.

Based on the evaluation of the morphological characteristics of the bulbs collected in the area of Premantura, they were classified as the sweet onion variety “Istarski crveni”(Istrian red), which is listed as a Protected Variety on the Variety List of the Republic of Croatia. However, given the specific agro-pedological conditions that prevail in the area of Premantura and the long tradition of cultivation, producers consider it a local landrace and specific product.

Onion bulb color was measured by a MiniScan^®^ EZ 4500 Portable Spectrophotometer (HunterLab, Reston, VA, USA). Color measurements were analyzed by considering the CIE (the Commission Internationale de l’Eclairage) L*a*b* color spaces. In the three-dimensional color space, L* represents lightness (pure white: 100%, pure black: 0%). The coordinate a* represents redness, i.e., the red to green axis, with positive a* being red, and negative a* green. Coordinate b*, i.e., yellowness, represents the yellow to blue axis with positive b* being yellow, and negative b* being blue. Color evaluation was performed on each bulb size group on different layers of the onion bulb. Firstly, the outer dry onion peel from each bulb was removed, put on white paper so that the rest of the onion bulb layers do not interfere with the color values due to the transparency of the dry layers, and captured with the device five times,. The first outer bulb flesh layer was measured two times for each bulb. The inner bulb flesh color was measured in horizontal cross section, three times for each bulb.

### 2.2. Sample Preparation

The selected bulbs were flash frozen in liquid nitrogen, lyophilized (Labogene Coolsafe 95-15 Pro, Allerød, Denmark), and milled to 0.2 mm with an ultra-centrifugal mill (Retsch ZM200, Haan, Germany). The extraction was performed according to Major et al. [36] by sonicating the ground sample (approximately 75 mg) in 80% aqueous methanol (MRC DCG-250H, Holon, Israel) for 30 min. The samples were macerated on an orbital shaker (GFL 3005, Lab Unlimited, Dublin, Ireland) at 25 °C and 150 rpm for 210 min. The samples were centrifuged at 16,000× *g* for 10 min (Domel Centric 350, Železniki, Slovenia) and subsequently filtered through a 0.22 µm nylon filter into HPLC vials. The extracts were kept at −80 °C until further analysis. Dry matter was determined gravimetrically in triplicate by drying the samples in an oven (Memmert UF160, Schwabach, Germany) at 105 °C until a consistent weight was obtained.

### 2.3. Total Phenolic Content and Total Antioxidant Capacity

The Folin–Ciocalteu assay [37] was used to determine the total phenolic content (TPC). Briefly, 20 µL of the sample was mixed with 140 µL of 0.2 M Folin–Ciocalteu reagent, and after 1 min 140 µL of 6% sodium carbonate was added. The mixture was kept at 25 °C for 60 min and the absorbance was read at 750 nm using a microplate reader (Tecan Infinite 200 Pro M Nano+, Männedorf, Switzerland). The results were expressed using a calibration curve with serial dilutions of gallic acid (12.5, 25, 50, 75, 100, 150, 250 mg/L; coefficient of determination, R^2^ = 0.9999) and expressed as mg GAE/g DW.

Total antioxidant activity was evaluated using the DPPH radical scavenging activity assay [38] and the FRAP assay [39]. Briefly, 100 µL of the sample were mixed with 200 µL of either freshly prepared 0.02M DPPH radical or FRAP reagent in a 96-well plate for the DPPH and FRAP assays, respectively. The DPPH radical scavenging capacity was determined after 30 min of reaction time at 25 °C by reading the absorbance at 517 nm on a microplate reader (Tecan Infinite 200 Pro M Nano+, Männedorf, Switzerland). The antioxidant capacity using the FRAP assay was evaluated after 10 min of reaction time at 25 °C by reading the absorbance at 593 nm on a microplate reader (Tecan Infinite 200 Pro M Nano+, Männedorf, Switzerland).

Both DPPH and FRAP values were calculated against a Trolox calibration curve (serial dilutions of Trolox—2, 5, 10, 25, 50, 75, 100 µM) and expressed as µmol TEQ/g DW.

### 2.4. Determination of the Onion Bulb Sugar Profile

The content of inulin, sucrose, glucose, and fructose in onion bulbs was determined by HPLC, which consisted of an autosampler (Shimadzu Nexera SIL-40CX3, Kyoto, Japan), a solvent delivery unit (Shimadzu Nexera LC-40DX3, Kyoto, Japan), a thermostatic column compartment (Shimadzu Nexera CTO-40C, Kyoto, Japan), and a refractive index detector (Shimadzu RID-20A, Kyoto, Japan). Sugar separation was achieved by injecting 10 µL of the sample into a calcium ion exchange column (300 × 8 mm, 9 µm particle size, Dr. Maisch ReproGel Ca, Ammerbuch, Germany) held at 80 °C using deionized water as the mobile phase (0.6 mL/min, isocratic elution). The identification and quantification of the investigated sugars were performed by comparing retention times and peak areas to analytical standards. The calibration curves were created by injecting serial dilutions of the investigated sugars (0.25, 0.50, 1.00, 2.50, 5.00, 7.50, and 10.00 g/L of inulin, sucrose, glucose, and fructose).

### 2.5. Determination of the Onion Bulb Phenolic Profile

The phenolic profile was analyzed on an LC-MS/MS, which consisted of an autosampler (Shimadzu Nexera SIL-40CX3, Kyoto Japan), two solvent delivery units (Shimadzu Nexera LC-40DX3, Kyoto, Japan), a thermostatic column compartment (Shimadzu Nexera CTO-40C, Kyoto, Japan) and a triple quadrupole mass spectrometer (Shimadzu LCMS8045, Kyoto, Japan). The separation was performed on a C18, 2.1 mm × 150 mm, 2.7 µm core–shell column (Advanced Materials Technology, Wilmington, DE, USA) held at 37 °C by injecting 1 µL of the sample using a linear gradient elution of mobile phase A (water/0.1% acetic acid) and mobile phase B (methanol/0.1% acetic acid) at 0.35 mL/minute, for 0 min to 0.75 min: 98%A; 0.75 min to 15 min: 98%A to 50%A; 15 min to 15.1 min: 50%A to 0%A; 15.1 min to 20 min: 0%A; 20 min to 20.1 min: 0%A to 98%A; and 20.1 min to 25 min: 98%A. The polyphenolic compounds were identified and quantified by using analytical standards, except for quercetin-3,7-diglucoside, and isorhamnetin-4′-glucoside which were tentatively identified by LC-MS/MS, using the characteristic precursor/product ions obtained by the fragmentation of quercetin-3,4′-glucoside and isorhamnetin-3-glucoside analytical standards, respectively. Quercetin-3,7,4′-triglucoside was identified using theoretical precusor/product ions.

### 2.6. Statistical Analysis

The analyses in this study were performed in three biological repetitions. The obtained data were analyzed by analysis of variance (ANOVA) and Partial Least Square Discriminant Analysis (PLS-DA) using Statistica 13.4 (Tibco, Inc, Palo Alto, CA, USA). Significant differences were determined at *p* ≤ 0.05 and homogenous group means were compared by Fischer’s Least Significant Difference post hoc test. The developed PLS-DA model was employed to investigate the discrimination power of individual phytochemical and nutritional parameters in onion bulb size differentiation.

## 3. Results

The sweet onion landrace “Premanturska kapula” was characterized by an average dry matter content of 7.33 ± 0.13 g/100 g of fresh bulb (Table 1). The average sugar content in bulb dry matter was 61.5 ± 1.7%. The most abundant sugar was glucose (28.0 ± 3.0 g/100 g DW), followed by fructose (21.2 ± 1.3 g/100 g DW), sucrose (10.2 ± 2.23 g/100 g DW), and finally fructooligosaccharides (2.09 ± 2.23 g/100 g DW).

The total bulb phenolic content was on average 3270 ± 327 µg GAE/g DW with an antioxidant capacity of 3.00 ± 0.66 µmol TE/g DW, 4.86 ± 0.50 µmol TE/g DW, and 79.8 ± 7.1 µmol TE/g DW for DPPH radical scavenging, FRAP, and ORAC, respectively.

The calculated sum of total phenolics from individual phenolic compounds was, on average, 4572 ± 703 µg/g DW with a minimum of 3587 and the maximum of 5392 µg/g DW. The most abundant flavonoid compounds were quercetin-3,4′diglucoside and quercetin-4′-glucoside with average values of 2272 ± 306 µg/g DW and 1210 ± 205 µg/g DW, respectively. Another important flavonoid compound in the investigated sweet onion landrace is isorhamnetin-4′-glucoside with an average content of 271 µg/g DW. The most abundant phenolic acid was protocatehuic acid with an average value of 729 ± 151 µg/g DW. Minor phenolic compounds detected in the investigated sweet onion cultivar were quercetin-3-glucoside (50.2 ± 10.2 µg/g DW), quercetin-3.7-diglucoside (26.4 ± 4.4 µg/g DW), vanillic acid (2.55 ± 0.44 µg/g DW), and quercetin (2.46 ± 0.35 µg/g DW).

The dry matter, total sugars, or bulb fructose content were not influenced by bulb size (Table 2). On the other hand, the analysis of variance showed a significant impact of bulb size on the sucrose, glucose, and fructooligosaccharide content. The highest sucrose content was observed in the large bulb size, followed by the medium bulb size, and finally, the lowest content was detected in the small onion bulb size. Fructooligosaccharide content was significantly higher in the large bulb size compared to the medium and small bulb size. On the other hand, the large bulb size had significantly lower glucose content compared to medium or small bulb size.

The total phenolic content was significantly higher in the medium compared to large or small bulb sizes, as was the antioxidant capacity measured by the DPPH radical scavenging assay. The FRAP or ORAC assays for antioxidant capacity did not show differences between bulb sizes.

The sum of the targeted phenolic compounds was, on the other hand, significantly lower in the large compared to medium and small bulb sizes, as was the case with the most abundant flavonoid compounds quercetin-3,4′-diglucoside and quercetin-4′-glucoside (Table 2). Other minor flavonoid compounds that were also significantly less abundant in the large compared to the medium or small bulb size were quercetin-3,7,4′-triglucoside and quercetin-3,7-diglucoside. The highest quercetin-3-glucoside content was detected in the medium bulb size, while the lowest was detected in the large bulb size. Isorhamnetin-4′-glucoside content was highest in the small bulb size, followed by the medium bulb size and then the large bulb size which had the lowest content. Protocatehuic acid content, as the most abundant phenolic acid, was also significantly lower in the large bulb size compared to the other sizes. Vanillic acid content was significantly lower in the large sized bulbs compared to the small sized bulbs.

The lightness (L) of the sweet onion bulb was significantly lower in the medium onion bulb size compared to the large or small bulb sizes (Table 3). The green to red ratio (a) was influenced by the interaction between bulb size and the bulb part wherein there were no differences in the inner bulb flesh color between bulb sizes; meanwhile, the outer bulb flesh color of the small and medium bulbs had a higher intensity red hue compared to the large bulb size. The green to yellow hue was influenced by both bulb size and bulb part. The large size bulb had a yellower hue compared to the medium and small sizes; meanwhile, the dry onion peel had the most intense yellow color regardless of bulb size (Table 3).

**Figure 1:**
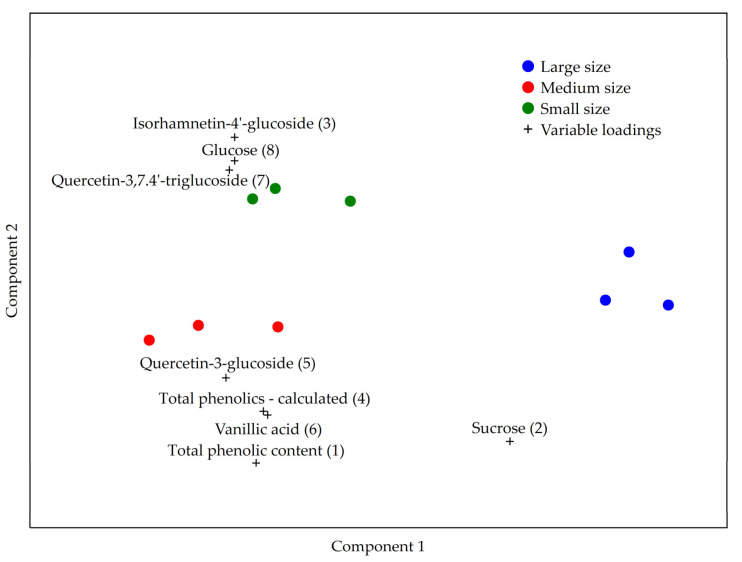
PLS-DA analysis of sweet onion “Premanturska kapula” bulb size.

The developed PLS-DA model showed that the most important parameters in the discrimination between the investigated bulb sizes in descending order of importance were total phenolic content, sucrose, isorhamnetin-4′-glucoside, sum of phenolics, quercetin-3-glucoside, vanillic acid, quercetin-3,7,4′-triglucoside, and glucose (Figure 1). The total phenolic content was used by the model to distinguish between medium versus small and large bulbs, while the sum of the investigated phenolics, vanillic acid, glucose, and quercetin-3,7,4′-triglucoside were used to distinguish between large versus small and medium sized bulbs. Sucrose, quercetin-3-glucoside, and isorhamnetin-4′-glucoside were used to distinguish between individual bulb size with each of them being characteristic for large, medium, and small sized bulb, respectively.

## 4. Discussion

There are many well-known sweet onion varieties and cultivars used in agriculture worldwide, such as the best-known variety “Vidalia Sweet Onion” and its cultivars “Nirvana”, “DPS 1032”, “Yellow 2025”, “king-Midas”, and “SBO 133” [20]. Other well-known varieties are “Sweet Georgia”, “Rio Bravo”, “Granex 33”, “Hybrid Yellow Granex”, “Dessex”, “Texas 1015Y”, “NUN 9746 F1”, Musica F1”, “Recorra F1”, “Cowboy F1 yellow”, “Domenica Supersweet”, and others [7].

Common onion bulbs have dry matter values ranging from 6 to 25%, with values lower than 15% being characteristic for sweet onion cultivars [40]. The Croatian local landrace sweet onion “Premanturska kapula” had lower dry matter content compared to the other known varieties and cultivars from various studies [20,41]. It is known that the water regime significantly affects the dry matter of onion; therefore, a good farming practice is prerequisite for optimal onion quality [40].

According to the USDA [42], sweet onion contains an average of 22.6 g of glucose, 20.2 g of fructose, 7.2 g of sucrose, and 9 g of fructooligosaccharides, resulting in a total sugar content of 59 g/100 g DW. The results of our study show that Croatian local landrace of sweet onion had, on average, higher sugar content than listed by the USDA, i.e., it contained 61.5 g/100 g DW of total sugar, which is composed of 28 g of glucose, 21.2 g of fructose, 10.2 g of sucrose, and 2.09 g of fructooligosaccharides. Fructans are hydrolyzed to fructose in cultivars with low dry matter content, which is important for osmoregulation. In cultivars with high dry matter content fructans are not hydrolyzed, this indicates that the most storable onion varieties have the lowest content of monosaccharides, while cultivars suitable for salads preparation are rich in glucose and fructose [40].

According to Loredana et al. [43], the four sweet onion varieties used in their study (Montoro onion, Alife onion, spinning-top Vatolla onion, and tapered shape Vatolla onion) had a total sugar content of 56.35 g/100 g DW which is lower compared to “Premanturska kapula”. According to Vavrina and Smittle [41], sweet onion cultivars such as “Sweet Georgia”, “Rio Bravo”, “Granex 33“, “Hybrid Yellow Granex”, “Dessex”, and “Texas 1015Y” had a total sugar content of 66.58 g/100g DW on average, with 33.45 g of glucose, 24.03 g of fructose, and 9.1 g of sucrose, i.e., a higher glucose and fructose content but a lower sucrose content compared to the landrace “Premanturska kapula”. According to Vågen and Slimestad, varieties such as “NUN 9746 F1”, “Musica F1”, “Recorra F1”, “Cowboy F1”, “Yellow Derby (BGS 220) F1”, “Domenica Supersweet”, “Colossus F1”, “Cronus F1”, “Cavalier F1”, “Summit F1”, and “Hytech F1” had an average total sugar content of 76.04 g/100 g DW, with on average 32.07 g of glucose, 25.52 g of fructose, 14.37 g of sucrose, and 4.07 g of fructooligosaccharides, i.e., 13% more glucose, 17% more fructose, 29% more sucrose, and 49% more fructooligosaccharides content than the Croatian local landrace “Premanturska kapula”.

The average total phenolic content obtained via colorimetric assay (TPC) for the “Premanturska kapula” was 3270 µg GAE/g DW. Several authors [20,43,44] reported higher total phenolic content in commercial sweet onion cultivars compared to “Premanturska kapula”. Sharma et al. [44] reported an average antioxidant capacity of 6.87 µmol TE/g DW and 12.58 µmol TE/g DW for DPPH and FRAP antioxidant capacity assays, respectively, in commercial sweet onion cultivars. The antioxidant capacity of “Premanturska kapula” was comparable to the results obtained by Ou et al. [45] who reported an average of 85 µmol TE/g DW for sweet white onions.

As the content of flavonoids in plants is affected by the combination of genotype, environmental conditions, and agronomic practices, different strategies can be applied in order to obtain foods enriched in antioxidant compounds, thus increasing their functional value [46]. Color is a phenotypical attribute that is closely related to the content in flavonoids in onions. Red cultivars generally contain higher flavonoid quantities than the white ones [47].

The identified flavonoids in our study are in line with the findings of Vågen and Slimestad [7], and Hedges and Lister [48], who state that onions contain flavonoids in various amounts, namely quercetin, isorhamnetin, and kaempferol and their derivatives. As shown by Golubkina and Carouso, onion ranks in first place for the concentration value of quercetin within a group of 28 vegetables and nine fruits [49] of which the antioxidant capacity is known to be the highest of the most common plant flavonoids [40]. Moreover, authors stated that at least eight flavonols are present in onions, such as quercetin-3-glucoside, quercetin-4′-glucoside, quercetin-7-glucoside, quercetin-3,4′-diglucoside, quercetin-7,4′-diglucoside, quercetin 3,7-diglucoside, quercetin 3,7,4′-triglucoside, and isorhamnetin-4′-glucoside, of which quercetin-4′-glucoside and quercetin-3,4′-diglucoside are considered to be the most abundant, accounting for more than 85% of total flavonol content in onions [7,48]. The remaining derivates are generally described as minor flavonols. In the study by Vågen and Slimestad [7] quercetin-3,4′-diglucoside ranged from 1160 (“Domenica Supersweet”) to 2610 µg/g DW (“Hytech F1”), with an average of 1776.36 µg/g DW. On average, “Premanturska kapula” exhibited 22% higher quercetin-3,4′-diglucoside content than the varieties used by Vågen and Slimestad [7]. The content of quercetin-3,4′-diglucoside of four sweet onion landraces (“Airola”, “Alife”, “Montoro”, “Vatolla”) used in the study by Cozzolino et al. [50] ranged from 537 (“Vatolla”) to 2260.7 µg/g DW (“Montoro), with an average of 1648.3 µg/g DW.

In the study by Vågen and Slimestad [7], the sweet onions contained an average of 2672.73 µg/g DW of quercetin-4′-glucoside, 349.09 µg/g DW of isorhamnetin-4′-glucoside, and 47.27 µg/g DW of quercetin-3-glucoside. The sweet onions investigated in the study by Cozzolino et al. [50] on average contained 1374.3 µg/g DW of quercetin-4′-glucoside, 213.4 µg/g DW isorhamnetin-4′-glucoside, and 37.4 µg/g DW of quercetin-3-glucoside.

Quercetin-3,7-diglucoside and quercetin-3,7.4′-triglucoside have been identified in onions and are considered to be minor flavonols [2,7,51,52,53]. Our results for quercetin-3,7.4′-triglucoside content are in line with the results of several authors [50,54,55,56].

Sharma et al. [44] reported an average of 11.03 µg/g DW of quercetin in sweet onion, which is a 78% higher content than what we quantified in the “Premanturska kapula”. In addition, Pinho et al. [56] reported an average content of quercetin aglycone of 21 µg/g DW, Vågen and Slimestad [7] reported an average of 190 µg/g DW, and Loredana et al. [43] reported an average of 381,4 µg/g DW.

Protocatehuic acid content for “Premanturska kapula” averaged at 729 µg/g DW, while the white onion variety “Armstrong” used in the study by Gorinstein et al. [57] averaged a significantly lower content of protocatehuic acid, namely 1.2 µg/g DW. According to the study by Gorinstein et al. [57], the white (sweet) onion “Armstrong” on average contained 6.3 µg/g DW of vanillic acid, which is a content 59% higher than what we quantified for the Croatian local landrace “Premanturska kapula”. Four sweet onion varieties (Montoro onion, Alife onion, spinning-top Vatolla onion tapered shape Vatolla onion) used in the study by Loredana et al. [43] had higher vanillic acid content compared to “Premanturska kapula”.

Sellapan and Akoh [20] reported 2957 µg/g DW of total phenolics in onions. According to Pinho et al. [56], the white onions used in their study on average contained 1870 µg/g DW of phenolics, which is lower compared to our results. An even lower content of total phenolics was reported by Loredana et al. [43] and Juániz et al. [58], at 1752.5 and 1360 µg/g DW, respectively. Although several individual phenolic compounds quantified in “Premanturska kapula” were found to be lower than what other authors reported, the total phenolics were found to be significantly higher in the landrace “Premanturska kapula” compared to other sweet onion varieties [20,43,56,58].

The maturity and bulb size is dependent on several factors, including the onion genotype, photoperiod sensitivity, planting density, nutrient and water availability, temperature, agronomic practices such as planting density or stalk removal, as well as the harvest time [28,59,60]. As the onion bulb matures, several changes take place, including changes in bulb size, composition, and flavor. These changes are driven by metabolic processes that influence the content and profile of both primary and secondary compounds present in onion bulbs [61].

During the early stages of onion bulb development, the primary source of carbon and energy for growth comes from leaf photosynthesis [62]. Sugars are subsequently transported to the bulb and stored as starch, but, as the bulb matures, the starch is gradually broken down into soluble sugars, thus increasing their concentration. Our results showed that there was no difference in dry matter content, as well as total soluble sugars or fructose content between different bulb sizes, thereby indicating a similar phenological stage. Dry matter content is closely linked to the genotype, but environmental factors such as water availability, temperature, light, and nutrient availability can have a significant impact on the accumulated dry matter [62]. On the other hand, significant differences were observed between glucose, sucrose, and fructooligosaccharide content among bulb sizes with the large bulb showing an increased fructooligosaccharide and sucrose content. Sinclair et al. observed a decrease in reducing sugars with the increase of soluble solids in onion bulbs and implied a possible increase in sugar polymerization [63]. Our results support this observation as the decrease in reducing sugars is not linked to decreasing fructose content but to the decrease in glucose content and an increase in sucrose and inulin-type fructooligosaccharides. Fructose to glucose content is a strong indicator of bulb maturity wherein decreasing levels indicate a more mature bulb. Benkeblia et al. studied the influence of temperature on sugar content during bulb maturation and observed an increase of sucrose with bulb maturity as well as temperature [64].

Onion bulbs are well known for their abundance in phytochemical compounds which have high antioxidant capacity. Our results showed that the antioxidant capacity measured by the DPPH radical scavenging assay, as well as all the investigated phenolic compounds except for quercetin and vanillic acid, were significantly lower in content on dry weight basis in the large compared to medium or small bulb size. Antioxidant capacity measured by ORAC and FRAP did not differ between onion bulb sizes. Lachowitz et al. investigated the antioxidant capacity of wild garlic and showed that although bulb total phenolic content increases with bulb maturity the antioxidant capacity is actually lower in more mature bulbs [65]. Lee et al. investigated the effect of conventional vs. organic farming on onion bulb yield, size, and nutritional quality where although conventional farming systems produce a larger bulb diameter the flavonoid content remains unchanged [66].

According to Golubkina and Caruso [40], genetic features seem to be affecting bulb quality the most, and to a greater extent the interactions between genotype, environment, and agricultural practices. Agricultural practices such as plant arrangement and density can affect bulb size [67]. Producers of the sweet onion landrace “Premanturska kapula“ traditionally use bare-root transplants for onion plantation establishment. Bare-root onion transplants, due to differences in seed size, germination speed, or seedlings plant density, are not uniform in size which may further affect plant development uniformity and, consequently, bulb size and yield [68]. The importance of transplants size on onion performance and bulb size was confirmed by several authors [69,70]. Generally, due to larger transplants increasing starting size and survivability, onion bulbs developed from such transplants can be of bigger size compared to those of smaller transplants. Additionally, due to traditional practices such as open pollination, a larger variability in bulb shape and size in traditional landraces such as “Premanturska kapula” is expected.

The difference in phenolic compounds content between large and smaller bulb size could be explained by the initial bioaccumulation of polyphenolic compounds in the bulb and the subsequent unequal development in bulb size. As shown by Cheng et al., the highest polyphenol content in both yellow and red onion cultivars is located in the older, outer bulb layers [71] indicating that the small and medium sized bulbs are of the same phenological stage as the larger bulb but with a difference in size. Our results showed that the outer bulb flesh layers were characterized by a more intense red hue compared to the inner layers, indicating a higher phenolic content. At the same time our results showed that the outer bulb flesh layers in medium and small bulb sizes had a significantly higher red hue compared to the large bulb size. The lower polyphenol content in the large size bulb could be a direct outcome of the faster growth rate and, consequently, the more rapid accumulation of dry matter because of various factors such as transplant size or agronomic practices. Nevertheless, the antioxidant properties of the investigated sweet onion landrace “Premanturska kapula” are excellent regardless of the bulb size choice.

## 5. Conclusions

Sweet onion varieties, known also as mild onions, are very popular for fresh consumption and the demand for these onions has increased in recent years. At the same time, the food market is constantly searching for new onion varieties, and domestic varieties have quality parameters that can meet consumer demand. The sweet onion landrace “Premanturska kapula” has a high soluble sugar content as well as a high antioxidant capacity and phenolic compound content compared to similar cultivars. Quercetin-3,4′-diglucoside and quercetin-4′-glucoside were the major flavonols, while protocatehuic acid was the major phenolic acid detected. The choice of onion bulb size can impact the profile of the sugars present, with the large bulb size favouring a higher sucrose and fructooligosaccharides content compared to small bulb sizes which were more abundant in glucose. However, the total sugars or bulb dry matter were not affected by bulb size. Phenolic compounds were more abundant in smaller bulb sizes, thus indicating a link between bulb development and phenolic compound allocation within the plant. From a consumer perspective it can be a choice between the small or medium bulb size, which are higher in polyphenolic content and simple sugars, or the larger sized bulbs which offer the same polyphenolic profile but are less abundant, with a reduced simple sugar content in favor for soluble fructooligosaccharides which are known to carry excellent health benefits.

## Figures and Tables

**Table 1:** Descriptive statistics of the sweet onion “Premanturska kapula” bulb nutritional parameters.

Parameter		Average	Minimum	Maximum	Standard Deviation
Dry matter	%	7.33	7.10	7.55	0.13
Soluble sugars					
Sum of sugars	g/100 g DW	61.5	59.4	64.6	1.7
Glucose		28.0	22.5	31.8	3.0
Fructose		21.2	18.6	23.0	1.3
Sucrose		10.2	7.6	13.4	2.3
Fructooligosaccharides		2.09	0.55	5.90	2.23
Total antioxidant capacity					
TPC	µg GAE/g DW	3270	2908	3767	327
DPPH	µmol TE/g DW	3.00	2.00	4.01	0.66
FRAP		4.86	4.20	5.57	0.50
ORAC		79.8	72.0	92.9	7.1
Phenolic compounds					
Total phenolics (calculated)	µg/g DW	4572	3587	5392	703
Quercetin-3,4′-diglucoside		2272	1907	2768	306
Quercetin-4′-glucoside		1210	932	1453	205
Protocatehuic acid		729	502	934	151
Isorhamnetin-4′-glucoside		271	176	342	62
Quercetin-3-glucoside		50.2	37.5	63.1	10.2
Quercetin-3,7-diglucoside		26.4	20.2	33.9	4.4
Quercetin-3,7.4′-triglucoside		9.1	7.6	10.6	1.1
Vanillic acid		2.55	2.03	3.28	0.44
Quercetin		2.46	1.85	2.89	0.35

**Table 2:** Differences in nutritional parameters between large, medium, and small sized bulbs in “Premanturska kapula” sweet onion landrace.

			Bulb Size		
Parameter		Large	Medium	Small	*p*-Value
Dry matter	%	7.4 ± 0.02	7.28 ± 0.04	7.3 ± 0.13	ns
Soluble sugars					
Total sugars	g/100 g DW	62.1 ± 1.4	61.6 ± 0.5	60.7 ± 1.2	ns
Glucose		24.4 ± 1.1 b ^1^	29.4 ± 0.5 a	30.4 ± 0.7 a	**
Fructose		19.8 ± 0.8	21.7 ± 0.3	22 ± 0.5	ns
Sucrose		12.9 ± 0.3 a	9.9 ± 0.3 b	7.7 ± 0.1 c	***
Fructooligosaccharides		5.02 ± 0.44 a	0.63 ± 0.07 b	0.64 ± 0 b	***
Total antioxidant capacity					
TPC	µg GAE/g DW	3011 ± 56 b	3684 ± 71 a	3116 ± 58 b	***
DPPH	µmol TE/g DW	2.25 ± 0.15 b	3.54 ± 0.24 a	3.21 ± 0.21 a	**
FRAP		4.41 ± 0.12	5.14 ± 0.29	5.04 ± 0.28	ns
ORAC		76.3 ± 2.9	83.8 ± 6.2	79.2 ± 2.7	ns
Phenolic compounds					
Total phenolics (calculated)	µg/g DW	3710 ± 84 b	5182 ± 160 a	4824 ± 191 a	**
Quercetin-3,4′-diglucoside		1924 ± 9 b	2525 ± 129 a	2366 ± 106 a	*
Quercetin-4′-glucoside		961 ± 24 b	1392 ± 56 a	1278 ± 49 a	**
Protocatehuic acid		556 ± 43 b	874 ± 44 a	756 ± 27 a	**
Isorhamnetin-4′-glucoside		196 ± 10 c	284 ± 12 b	332 ± 9 a	***
Quercetin-3-glucoside		39.0 ± 1.3 c	61.9 ± 0.9 a	49.6 ± 2.1 b	***
Quercetin-3,7-diglucoside		21.6 ± 0.8 b	29.7 ± 2.5 a	27.9 ± 1.2 a	*
Quercetin-3,7.4′-triglucoside		7.81 ± 0.10 b	9.55 ± 0.42 a	9.97 ± 0.35 a	*
Vanillic acid		2.21 ± 0.10 b	3 ± 0.26 a	2.44 ± 0.11 ab	*
Quercetin		2.52 ± 0.21	2.34 ± 0.24	2.52 ± 0.22	ns

ns—not significant; * *p* ≤ 0.05; ** *p* ≤ 0.01; *** *p* ≤ 0.001; ^1^ different letters indicate different groups in Fisher’s Least Significant Difference test.

**Table 3:** LAB color space values for the sweet onion landrace “Premanturska kapula” depending on bulb size and bulb part.

		*L*	*a*	*b*
Bulb size	Small	58.3 ± 1.5 a ^1^	7.3 ± 1 b	4.4 ± 1.2 b
	Medium	53.2 ± 1.4 b	9.2 ± 1.3 a	3.6 ± 1.3 b
	Large	61.0 ± 1.1 a	6.0 ± 1.0 b	6.7 ± 1.1 a
	*p*-value	**	***	***
Bulb part	Outer bulb flesh	58.0 ± 1.2	11.6 ± 0.7 a	−0.2 ± 0.9 c
	Inner bulb flesh	58.6 ± 1.3	2.7 ± 0.2 b	4.3 ± 0.3 b
	Outer dry peel	54.2 ± 2.2	12.6 ± 1.3 a	14.3 ± 1 a
	*p*-value	ns	***	***
Bulb size × Bulb part				
Inner bulb flesh	Small	60.7 ± 1.8	3.0 ± 0.3 d	4.5 ± 0.7
	Medium	53.3 ± 2.3	3.3 ± 0.4 d	3.1 ± 0.5
	Large	61.7 ± 1.7	1.7 ± 0.3 d	5.4 ± 0.4
Outer bulb flesh	Small	55.2 ± 2.3	12.5 ± 1 b	-0.6 ± 2
	Medium	55.5 ± 1.3	13.8 ± 1.2 ab	-2.5 ± 0.9
	Large	63.3 ± 1.1	8.3 ± 0.4 c	2.6 ± 0.8
Outer dry peel	Small	57.4 ± 5.2	9.1 ± 2.5 c	11.8 ± 2.1
	Medium	49.4 ± 3.4	16.1 ± 1 a	14.5 ± 0.9
	Large	55.9 ± 2.3	12.7 ± 2.2 b	16.7 ± 1.6
	*p*-value	ns	***	ns

*L*—lightness; *a*—red to green hue; *b*—blue to yellow hue; ns—not significant; ** *p* ≤ 0.01; *** *p* ≤ 0.001; ^1^ different letters indicate different groups in Fisher’s Least Significant Difference test.

## Data Availability

All the data is contained in this article.
